# Receptor Activity Modifying Protein RAMP Sub-Isoforms and Their Functional Differentiation, Which Regulates Functional Diversity of Adrenomedullin

**DOI:** 10.3390/biology11050788

**Published:** 2022-05-21

**Authors:** Takayuki Shindo, Megumu Tanaka, Akiko Kamiyoshi, Yuka Ichikawa-Shindo, Hisaka Kawate, Takayuki Sakurai

**Affiliations:** 1 Department of Cardiovascular Research, Shinshu University School of Medicine, Matsumoto 390-8621, Japan; uvlc1sarn3qv@yahoo.co.jp (M.T.); kamiyosh@shinshu-u.ac.jp (A.K.); yshindo@shinshu-u.ac.jp (Y.I.-S.); hkawate@shinshu-u.ac.jp (H.K.); tsakurai@shinshu-u.ac.jp (T.S.); 2 Department of Life Innovation, Institute for Biomedical Sciences, Interdisciplinary Cluster for Cutting Edge Research, Shinshu University, Matsumoto 390-8621, Japan

**Keywords:** adrenomedullin, RAMP, genetically engineered mice, cardiovascular diseases, cancer metastasis

## Abstract

**Simple Summary:**

The body contains a variety of humoral bioactive factors and information regulation systems that maintain homeostasis. Although adrenomedullin (AM) was first identified as a vasodilator peptide, subsequent studies have revealed that it plays an essential role in homeostasis in vivo and possesses a variety of bioactivities other than vasodilation. Based on its structural homology, AM is classified as a member of the calcitonin superfamily, which includes calcitonin, calcitonin gene-related peptide (CGRP), amylin, and intermedin. Peptides belonging to the calcitonin superfamily are characterized by a partial sharing of their receptor system. One of the sub-isoforms of receptor activity-modifying protein (RAMP) binds to calcitonin receptor-like receptor (CLR), a 7-transmembrane G protein, in a one-to-one manner, and then functions as a receptor for these peptides. Among RAMP isoforms, we focused on RAMP2 and RAMP3. Each RAMP isoform is expected to be a potential therapeutic target for various diseases, including cardiovascular diseases and cancer metastasis. In this review, we outline the pathophysiological significance and functional differentiation of RAMP2 and RAMP3, as mainly revealed by the analysis of genetically engineered mice.

**Abstract:**

AM knockout (AM-/-) and RAMP2 knockout (RAMP2-/-) proved lethal for mice due to impaired embryonic vascular development. Although most vascular endothelial cell-specific RAMP2 knockout (E-RAMP2-/-) mice also died during the perinatal period, a few E-RAMP2-/- mice reached adulthood. Adult E-RAMP2-/- mice developed spontaneous organ damage associated with vascular injury. In contrast, adult RAMP3 knockout (RAMP3-/-) mice showed exacerbated postoperative lymphedema with abnormal lymphatic drainage. Thus, RAMP2 is essential for vascular development and homeostasis and RAMP3 is essential for lymphatic vessel function. Cardiac myocyte-specific RAMP2 knockout mice showed early onset of heart failure as well as abnormal mitochondrial morphology and function, whereas RAMP3-/- mice exhibited abnormal cardiac lymphatics and a delayed onset of heart failure. Thus, RAMP2 is essential for maintaining cardiac mitochondrial function, while RAMP3 is essential for cardiac lymphangiogenesis. Transplantation of cancer cells into drug-inducible vascular endothelial cell-specific RAMP2 knockout mice resulted in enhanced metastasis to distant organs, whereas metastasis was suppressed in RAMP3-/- mice. RAMP2 suppresses cancer metastasis by maintaining vascular homeostasis and inhibiting vascular inflammation and pre-metastatic niche formation, while RAMP3 promotes cancer metastasis via malignant transformation of cancer-associated fibroblasts. Focusing on the diverse physiological functions of AM and the functional differentiation of RAMP2 and RAMP3 may lead to the development of novel therapeutic strategies.

## 1. Introduction

The body maintains homeostasis via an elaborate system of signaling factors and information regulation systems, the dysfunction of which may lead to the development of various diseases. Humoral bioactive factors, which are produced in various cells and organs throughout the body, act as signaling factors that play a role in the maintenance of homeostasis and inter-organ coordination. Adrenomedullin (AM), a bioactive peptide found in human pheochromocytoma, consists of 52 amino acids and is characterized by a ring structure composed of six amino acids within the molecule and an amide group at the C-terminus [1]. Initially, AM was described as a vasodilator, but subsequent studies indicate that it exerts a variety of physiological effects other than vasodilation [2]. AM is widely distributed in vivo and exerts various effects, such as inhibition of endothelial cell apoptosis, anti-atherosclerosis, angiogenesis, increased coronary blood flow, inhibition of cardiac hypertrophy and fibrosis in the heart, bronchodilation in the lung, increased renal blood flow, and diuretic effects in the kidney. Recently, it was reported that administering AM to patients with inflammatory bowel disease led to remission of symptoms, subsequent to which clinical application of AM is expected [3,4].

However, the short half-life of AM and other bioactive peptides in blood limits their application as therapeutic agents for chronic diseases. Therefore, we focused on the AM receptor system. AM is considered to be closely related to calcitonin, amylin, and calcitonin gene-related peptide (CGRP) in molecular evolutionary terms, these peptides being considered as members of the calcitonin superfamily. Peptides belonging to the calcitonin superfamily partially share receptor system. One of the sub-isoforms of receptor activity-modifying protein (RAMP) binds to calcitonin receptor-like receptor (CLR), a 7-transmembrane G protein, in a one-to-one manner, and subsequently functions as a receptor for these peptides. It is well known that the combination of CLR and RAMP1 shows high affinity for CGRP, whereas the combination of CLR and RAMP2 or RAMP3 shows high affinity for AM [5]. Amino acid homology between RAMP isoforms is low at approximately 30%. Each RAMP is characterized by a long extracellular domain that is thought to interact with the extracellular domain of CLR and regulate its binding affinity with these peptides. All RAMP isoforms are widely distributed in vivo.

In this review, we focus on RAMPs, especially RAMP2 and RAMP3, which regulate the diverse physiological functions of AM as well as members of its peptide family, and outline their functional differentiation, mainly based on the analysis of genetically engineered mice.

## 2. Results

### 2.1. Regulation of Vascular Homeostasis by the AM-RAMP2 System

Although AM was originally identified as a vasodilator, we found that homozygous AM knockout (AM-/-) mice had immature vascular development and major abnormalities in the structure of vascular endothelial cells, resulting in embryonic lethality with diffuse bleeding and systemic edema at mid-gestation [6]. The mortality rate among AM-/- embryos at embryonic day (E) 13.5 was 83%, and none survived to E14.5. At E12.5, before the hemorrhagic changes were detectable macroscopically, endothelial cells in AM-/- embryos had partially detached from the basement membrane within vessels, and the three-layer structure of the basal membrane was not clearly recognizable. These changes likely contributed to the fragility of the vascular wall and subsequent hemorrhage and edema. These results indicate that AM is essential for vascular development. 

On the other hand, since the expression of RAMP2, among the RAMP sub-isoforms, was upregulated in the lethal stage of AM-/- embryos, we hypothesized that RAMP2 may be important in the regulation of AM function in blood vessels. Therefore, we established RAMP2 knockout (RAMP2-/-) mice and determined that RAMP2-/- mice were embryonically lethal due to the same vascular developmental defects as AM-/- mice [7]. Our finding that RAMP2-/- mice carry a lethal phenotype similar to that of AM-/- mice suggests that the AM-RAMP2 system is specifically be involved in vascular development. The absence of changes in the expression levels of other RAMP sub-isoforms indicates an absence of complementarity between RAMP sub-isoforms during development and that the AM-RAMP2 system is essential for normal vascular development.

To directly clarify the physiological functions of the vascular AM-RAMP2 system, we generated vascular endothelial cell-specific RAMP2 knockout (E-RAMP2-/-) mice by crossbreeding RAMP2 flox/flox mice and VE-cadherin-Cre transgenic mice [8]. Whereas conventional RAMP2-/- embryos died at mid-gestation, E-RAMP2-/- embryos survived until later development, although most died during the perinatal period. Perinatal E-RAMP2-/- mice exhibited systemic edema and vascular abnormalities, including malformation of endothelial cells with partial detachment from the basement membrane. Unlike those in conventional RAMP2-/- embryos, the lesions in E-RAMP2-/- embryos were limited to endothelial cells, which likely accounted for their longer survival. Nonetheless, most E-RAMP2-/- mice died due to edema, reflecting endothelial abnormalities and the resultant vascular leakage. Thus, endogenous RAMP2 appears to be essential for endothelial cell viability and vascular integrity.

In conditional targeting in E-RAMP2-/- mice, penetration of RAMP2 gene deficiency was not 100%. Among the 5% of E-RAMP2-/- mice that survived until adulthood, the remaining RAMP2 expression in endothelial cells was approximately 20% of that in littermate controls. In the surviving E-RAMP2-/- adults, the aortic smooth muscle layers were disarrayed and the endothelial cells were detached from the basement membrane and severely deformed. These phenomena suggest that congenital endothelial cell abnormalities may induce a secondary postnatal disorder that affects other vascular component cells. Furthermore, E-RAMP2-/- adults showed a marked accumulation of inflammatory cells along the blood vessels within major organs and chronic vascular damage due to exacerbated inflammation. Interestingly, organ damage, such as cirrhosis-like changes, cardiac enlargement and fibrosis, hydronephrosis, and renal polycystic changes, were all found to spontaneously develop in E-RAMP2-/- adults with aging. These findings suggest that endothelial RAMP2 deficiency is the primary cause of subsequent vascular inflammation, fibrosis, and chronic organ dysfunction. 

Because a limited number of E-RAMP2-/- adults were available, we next generated drug-inducible vascular endothelial cell-specific RAMP2 knockout (DI-E-RAMP2-/-) mice, in which RAMP2 deficiency could be induced in adults [8]. Mice expressing tamoxifen-inducible Cre-recombinase under the regulation of VE-cadherin promoter were crossed with RAMP2 flox/flox mice to create tamoxifen-inducible DI-E-RAMP2-/- mice. In DI-E-RAMP2-/- mice, the development of systemic edema associated with increased vascular permeability was observed early after the induction of RAMP2 gene deletion, even in adults. Impaired polymerization of actin as well as particularly decreased formation of cortical actin rings (actin-bundle formation under the plasma membrane) was observed in vascular endothelial cells. It has been reported that small GTPases, Rac1 and RhoA, play crucial roles in the regulation of the barrier function of endothelial cells by regulating the formation of cortical actin rings and stress fibers, respectively [9]. We found that Rac1 activity was decreased and RhoA activity was increased in endothelial cells primarily cultured from DI-E-RAMP2-/-. Conversely, the formation of cortical actin rings in wild-type endothelial cells was enhanced by the addition of AM. Thus, the AM-RAMP2 system upregulates cAMP and leads to Rac1 activation, which in turn strengthens endothelial barrier function by enhancing cortical actin rings. These results emphasize the importance of the AM-RAMP2 system in maintaining vascular endothelial cell morphology.

**Figure 1:**
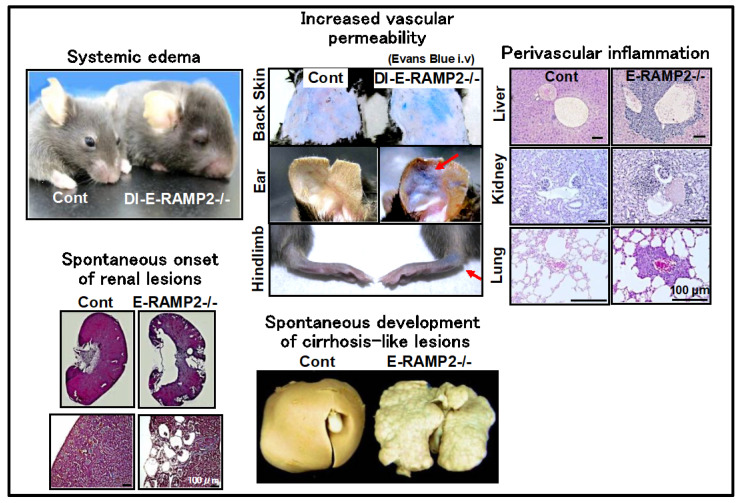
Phenotypes of vascular endothelial cell-specific RAMP2 knockout mice.

Next, we induced wire injury in the femoral arteries of DI-E-RAMP2-/- mice to examine neointima formation. After 4 weeks, neointima formation was markedly enhanced in DI-E-RAMP2-/- mice, accompanied by suppression of re-endothelialization, increased smooth muscle proliferation, increased macrophage infiltration of the neointima and adventitia, and increased oxidative stress levels [10]. These results indicate that the vascular AM-RAMP2 system is essential not only for vascular development, but also for vascular homeostasis in adults (Figure 1).

The phenotypes of vascular endothelial cell-specific RAMP2 knockout (E-RAMP2-/-) mice and drug-inducible vascular endothelial cell-specific RAMP2 knockout (DI-E-RAMP2-/-) mice are shown. E-RAMP2-/- and DI-E-RAMP2-/- mice have impaired vascular homeostasis and spontaneously develop increased vascular permeability, systemic edema, perivascular inflammation, and damage to various organs.

### 2.2. Functional Differentiation of AM-RAMP2 and AM-RAMP3 Systems in the Regulation of Vascular Homeostasis

Unlike RAMP2-/- mice, RAMP3 knockout (RAMP3-/-) mice showed no abnormalities in vascular development, and adults were obtained. When angiogenic potential was evaluated in adults using a unilateral hind limb ischemia model, no changes were observed in RAMP3-/- mice compared to wild-type mice, indicating that RAMP3 is not involved in angiogenesis, not only during development, but also during adulthood [11].

RAMP2 is highly expressed in vascular endothelial cells, whereas RAMP3 is highly expressed in lymphatic endothelial cells. To evaluate lymphatic vessels in RAMP3-/- mice, we performed fluorescence lymphography using indocyanine green (ICG), which is used clinically to assess lymphatic drainage function. We found that ICG drainage via subcutaneous lymphatic vessels was delayed in RAMP3-/- mice compared to that in wild-type mice. Similarly, chyle transport by intestinal mesenteric lymphatics was also delayed in RAMP3-/- mice fed a high-fat meal.

Next, we examined a postoperative lymphedema model. Tail skin contains a highly regular network of lymphatic vessels. Therefore, circumferential removal of the skin from the tail obstructs lymphatic flow, resulting in acute lymphedema and enabling this system to be used as a model of postoperative lymphedema. We found that compared to wild-type mice, both vascular and lymph vessel-specific RAMP2-/- mice did not show changes in the severity of postoperative lymphedema. However, only RAMP3-/- mice showed worsening of edema. Although there was no change in the number of neoplastic lymph vessels or blood vessels in RAMP3-/- mice, abnormal dilation of lymph vessels, exacerbation of interstitial edema, and increased inflammatory cell infiltration were observed. Electron microscopy showed characteristic vacuolar degeneration and failed anchoring filament formation in lymphatic endothelial cells of RAMP3-/- mice. Primary cultures of lymphatic endothelial cells from RAMP3-/- embryos were examined, and showed reduced migration ability as well as reduced activity of Akt, a cell survival signaling factor.

**Figure 2:**
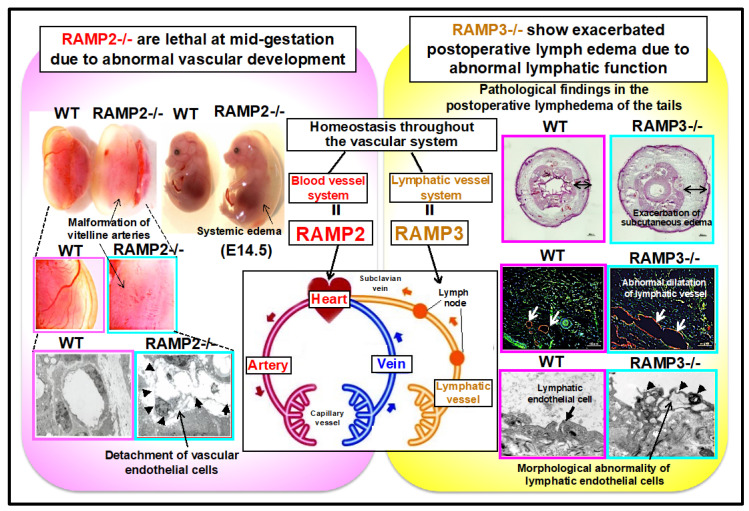
Functional differentiation of AM-RAMP2 and AM-RAMP3 systems in the regulation of vascular homeostasis.

These results indicate that the AM-RAMP2 system regulates angiogenesis during development and vascular homeostasis in adults, whereas the AM-RAMP3 system regulates lymphatic function in adults. The AM-RAMP system regulates the homeostasis of the entire vascular system, composed of blood and lymphatic vessels, via the functional differentiation of RAMP2 and RAMP3 (Figure 2).

Phenotypes of RAMP2 knockout (RAMP2-/-) mice and RAMP3 knockout (RAMP3-/-) mice are shown. RAMP2-/- mice are embryonically lethal at mid-gestation, owing to abnormal vascular development. Although RAMP3-/- mice do not show developmental abnormalities, adults show lymphatic dysfunction and exacerbated postoperative lymphedema.

### 2.3. Functional Differentiation of AM-RAMP2 and AM-RAMP3 Systems in Cardiovascular Stress Response

In addition to being expressed in the vascular system, RAMP2 and RAMP3 are also highly expressed in the heart. Induction of RAMP2 deficiency in adult mice resulted in the spontaneous onset of a dilated cardiomyopathy-like finding which was lethal [12]. By contrast, congenic cardiac myocyte-specific RAMP2 knockout mice (C-RAMP2-/-) were born normally and showed no apparent changes in steady-state cardiac function. Therefore, we examined the cardiovascular stress response in C-RAMP2-/- mice using transverse aortic constriction (TAC) surgery [13]. Compared to the controls, C-RAMP2-/- mice displayed impaired cardiac function, increased cardiac hypertrophy and fibrosis, and decreased survival, as early as on day 7 after TAC.

Electron microscopic observations revealed that even without TAC, C-RAMP2-/- cardiac myocytes exhibited myofibrillar disarray and various mitochondrial structural abnormalities (dropout, swelling, deformation of cristae, etc.). Using a Ca^2+^ imaging system to analyze twitch Ca^2+^ transients in Fluo-4-loaded cardiac myocytes, we determined that the peak Ca^2+^ response (△F/F0), defined as the ratio of peak cytosolic Ca^2+^ fluorescence elicited by electric pacing (△F) to basal cytosolic Ca^2+^ fluorescence without stimulation (F0), was significantly lower in C-RAMP2-/- than in wild-type cardiac myocytes. These observations indicate the presence of latent dysfunction in C-RAMP2-/- cardiac myocytes.

Using primary cultured cardiac myocytes isolated from C-RAMP2-/- mice, we assessed the response to a β-receptor agonist, isoproterenol (ISO). A fluorescent probe which stains the mitochondria, MitoBright green, showed that the distribution of viable mitochondria was significantly reduced by ISO treatment in C-RAMP2-/- cardiac myocytes. At the same time, ISO treatment significantly reduced the mitochondrial membrane potential indicator, JC-1 red/green ratio, in C-RAMP2-/- cardiomyocytes, indicating a marked reduction in mitochondrial membrane potential. Mitochondrial respiration analysis (Mito stress test) showed that both mitochondrial respiration capacity and ATP production were lower in C-RAMP2-/- cells than in wild-type cells. These results indicate that the absence of RAMP2 may lead to mitochondrial dysfunction in cardiomyocytes.

In C-RAMP2-/- cardiac myocytes, ISO treatment downregulated the expression of PGC-1α and PGC-1β, two master regulators of mitochondrial biogenesis. The level of cAMP response element binding (CREB) protein activation was also reduced in C-RAMP2-/- cardiac myocytes. CREB is known to upregulate the expression of PGC-1α and PGC-1β [14], and thus downregulation of CREB activation may underlie observed cardiac mitochondrial dysfunctions in C-RAMP2-/- mice.

Considering the marked changes observed in cardiac structure and function induced by TAC in C-RAMP2-/- mice, the effects of TAC on RAMP3-/- mice were comparatively mild. In fact, we detected no changes in the cardiac mitochondria in RAMP3-/- mice. However, after 4 weeks of TAC, there was a significant reduction in cardiac function with enhanced fibrosis in RAMP3-/- mice compared to their wild-type littermates. In order to clarify the mechanism underlying the milder and slower response to TAC in RAMP3-/- mice, we focused on lymphatic vessels in the heart. To visualize cardiac lymphatic vessels, we immunostained sections of RAMP3-/- hearts for the lymphatic vessel marker LYVE-1. Staining revealed a significantly greater reduction in cardiac lymphatic vessels after 4 weeks of TAC in RAMP3-/- mice. AM was previously reported as regulating the expression of a gap junction protein, connexin 43 (Cx43) in lymphatic endothelial cells and also promoting cardiac lymphangiogenesis [15,16]. Therefore, we next analyzed Cx43 distribution in heart sections and found that Cx43-immunostaining was reduced in TAC-operated RAMP3-/- mice. Cx43 is thought to be essential for lymphatic drainage function, and RAMP3-/- mice showed particularly poor lymphatic vessel development around the coronary arteries, suggesting that chronic inflammation due to impaired drainage of tissue fluid persisted, leading to the formation of characteristic fibrotic lesions around the coronary arteries.

**Figure 3:**
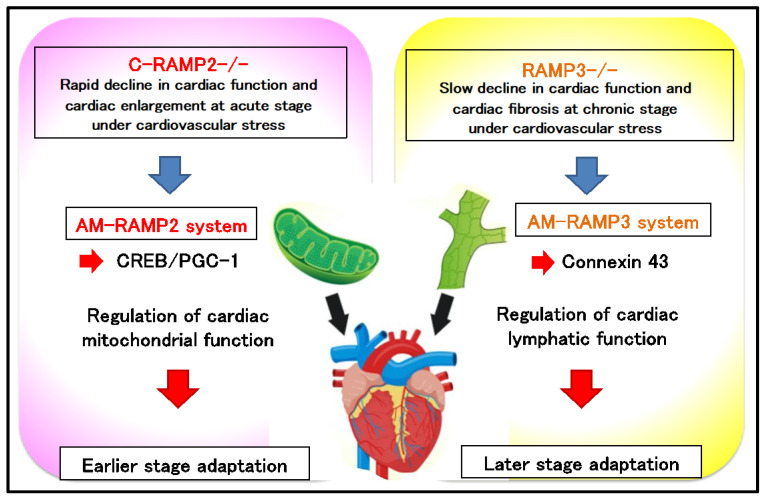
Functional differentiation of AM-RAMP2 and AM-RAMP3 systems in response to cardiovascular stress.

These results indicate that the AM-RAMP2 system is essential for maintaining mitochondrial function in cardiac myocytes, and that it is required early in the cardiovascular stress response. By contrast, the AM-RAMP3 system is required for stress response at a later stage through the regulation of lymphatic homeostasis (Figure 3).

A single intravenous injection of adenoviral vectors harboring the human AM gene showed positive effects on cardiac function and pathology in several experimental models of hypertension, diabetes mellitus, and myocardial infarction [17]. How RAMP2 and RAMP3 are related to the pathogenesis of these various heart diseases requires further investigation.

Transverse aortic constriction (TAC) surgery results in rapid cardiac dysfunction and cardiac enlargement in cardiac myocyte-specific RAMP2 knockout (C-RAMP2-/-) mice, whereas in RAMP3-knockout (RAMP3-/-) mice, it results in chronic phase cardiac dysfunction and fibrosis. The AM-RAMP2 system regulates cardiac mitochondrial homeostasis via CREB-PGC-1 signaling, whereas the AM-RAMP3 system regulates cardiac lymphatic function by regulating connexin 43 (Cx43). The AM-RAMP2 system is necessary for acute adaptation to cardiovascular stress, whereas the AM-RAMP3 system is necessary for chronic adaptation.

### 2.4. Regulation of Vascular Homeostasis and Suppression of Cancer Metastasis by the AM-RAMP2 System

AM and its receptor system are reportedly expressed in many cancers, including lung, breast, and pancreatic [18,19,20]. We investigated the association between the AM-RAMP2 system in blood vessels and cancer growth and metastasis, using DI-E-RAMP2-/- mice to deplete RAMP2 in the vascular endothelial cells of adults [21].

Subcutaneous transplantation of B16F10 melanoma cells into DI-E-RAMP2-/- mice resulted in attenuated intra-tumoral angiogenesis and suppressed tumor growth compared to control mice. This result suggests that RAMP2 plays a critical role in angiogenesis of the primary lesion and that RAMP2-deletion suppresses tumor growth.

**Figure 4:**
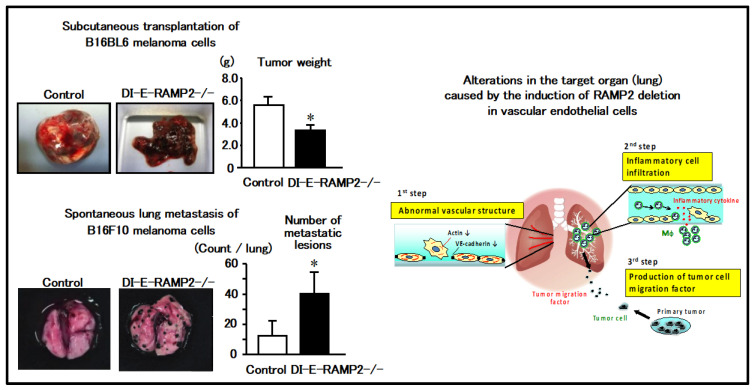
Regulation of vascular homeostasis and inhibition of cancer metastasis by the AM-RAMP2 system. * represents *p* < 0.05.

In contrast to B16F10 melanoma cells, subcutaneously implanted B16BL6 melanoma cells spontaneously metastasize to the lungs. To assess the effect of RAMP2 deletion on tumor metastasis, B16BL6 melanoma cells were transplanted into the hindlimb footpads of DI-E-RAMP2-/- and control mice. Three weeks later, the primary tumors were resected, and analyzed for spontaneous lung metastasis 4 weeks following resection. Contrary to our expectations, the incidence of metastasis and the number of metastatic lesions were higher in DI-E-RAMP2-/- mice than in control mice (Figure 4; left). Thus, DI-E-RAMP2-/- mice had smaller primary tumors, but more metastasis to distant organs, a seemingly contradictory result.

To clarify the mechanism underlying enhanced metastasis in DI-E-RAMP2-/- mice, we investigated vascular alterations in DI-E-RAMP2-/- mice following transplantation of cancer cells. Endothelial-mesenchymal transition (EndMT), which destabilizes vascular structures and promotes cancer invasion and metastasis, has attracted considerable attention [22]. Within the primary lesions of DI-E-RAMP2-/- mice, abnormal overgrowth of αSMA-positive mesenchymal cells in the vascular walls was accompanied by a reduction in the number of CD31-positive endothelial cells, suggesting the occurrence of EndMT.

Next, we observed changes in the lungs, which are the target organs for metastasis. Recently, it was suggested that a favorable environment within a distant organ increases the likelihood of cancer metastasis, leading to the concept of pre-metastatic niches [23,24,25]. Electron microscopy revealed that the pulmonary endothelial cells in DI-E-RAMP2-/- mice were deformed. Moreover, we detected a prominent invasion of macrophages, which were detected mainly around the vasculature, as well as an increased expression of proinflammatory cytokines during the early stages of tumor metastasis. Inflammation persisted even after RAMP2 gene deficiency was induced, and prior to tumor metastasis we observed increased expression of S100A8/A9 and its downstream factor, SAA3, which are known to recruit tumor cells to metastatic sites [23,24], suggesting that they may promote pre-metastatic niche formation in the target organ (Figure 4; right).

By contrast, tumor cell adhesion to vascular endothelial cells and metastasis to distant organs were suppressed in mice overexpressing RAMP2, which improved their survival rates, indicating that activation of the AM-RAMP2 system suppresses cancer metastasis. Thus, regulation of vascular homeostasis via the AM-RAMP2 system may lead to therapies that inhibit not only cardiovascular diseases, but also cancer metastasis.

(Left) Transplantation of melanoma cells into drug-inducible vascular endothelial cell-specific RAMP2 knockout (DI-E-RAMP2-/-) mice suppresses local growth, whereas metastasis to distant organs is enhanced. (Right) DI-E-RAMP2-/- mice show inflammatory cell infiltration of the vessel wall along with abnormal vascular structures in the lung. Inflammatory cells infiltrating the vascular wall produce various inflammatory cytokines and tumor migration factors, forming a pre-metastatic niche. As a result, DI-E-RAMP2-/- mice show increased metastasis to distant organs, even though the primary lesions are small.

### 2.5. Antagonism between AM-RAMP2 and AM-RAMP3 Systems in Cancer Metastasis

Pancreatic cancer has a poor prognosis, wherein regulation of liver metastasis becomes a particularly important issue. Next, we conducted liver metastasis experiments on pancreatic cancer cells using DI-E-RAMP2-/- mice [26]. Pan02 pancreatic cancer cells were transplanted into the spleen, following which metastasis to the liver was examined. DI-E-RAMP2-/- mice showed an increased metastasis rate compared with the control group. This result was similar to that mentioned above, which indicated that lung metastasis of melanoma cells was enhanced in DI-E-RAMP2-/- mice. Furthermore, we found that the expression of podoplanin (PDPN), a known marker of lymphatic vessels as well as cancer-associated fibroblasts (CAF), was markedly upregulated in the metastatic lesions of DI-E-RAMP2-/- mice. PDPN-positive CAFs in pancreatic cancer are reportedly associated with a poor prognosis [27]. In fact, PDPN-positive CAFs were increased in the metastatic lesions of DI-E-RAMP2-/- mice, indicating its involvement in enhancing liver metastasis.

The expression of RAMP3 was increased while the expression of RAMP2 was decreased in the metastatic lesions of DI-E-RAMP2-/- mice. To elucidate the role of elevated RAMP3 expression, we examined the liver metastasis model using RAMP3-/- mice [26]. Interestingly, in contrast to DI-E-RAMP2-/- mice, liver metastasis was suppressed in RAMP3-/- mice. Furthermore, in contrast to DI-E-RAMP2-/- mice, PDPN-positive CAFs at metastatic sites in the periphery of tumors were found to be reduced in RAMP3-/- mice.

RAMP3 knock down in mouse embryonic fibroblasts caused a decrease in PDPN expression, confirming the presence of PDPN downstream of RAMP3. PDPN reportedly exerts effects on downstream of Src and Cas by activating RhoA, which is involved in cytoskeletal remodeling and epithelial-mesenchymal transition (EMT) [28]. The expression and activity of Src and Cas were also downregulated in RAMP3-/- CAFs. These results indicate that Src-Cas-PDPN-RhoA signaling occurs downstream of RAMP3. Furthermore, in RAMP3-/- CAFs, cytoplasmic stress fiber formation was suppressed, while cortical actin ring formation under the plasma membrane was enhanced, suggesting mesenchymal-epithelial transition (MET). RAMP3-/- CAFs inhibited the proliferation and migration of Pan02 pancreatic cancer cells when co-cultured with Pan02 cells. Furthermore, the expression levels of tumor growth-promoting factors were decreased, while the expression levels of tumor growth inhibitory factors were increased, in RAMP3-/- CAFs. In fact, subcutaneous transplantation of RAMP3-/- CAFs mixed with Pan02 cells into mice inhibited tumor growth.

**Figure 5:**
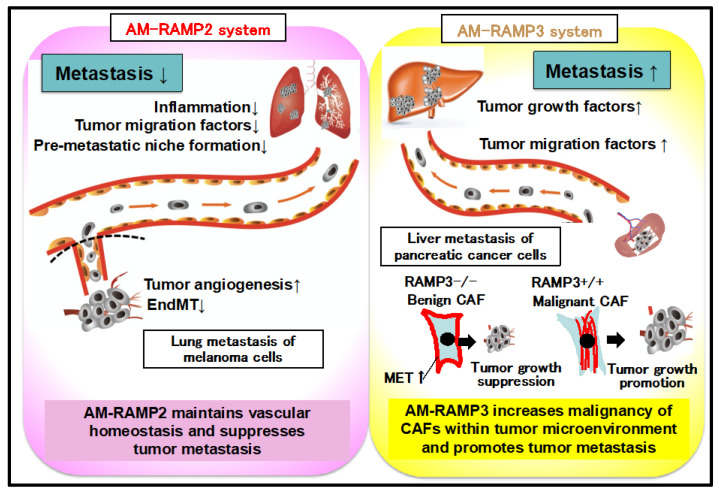
Antagonistic effects of AM-RAMP2 and AM-RAMP3 systems on cancer metastasis.

These results suggest that the malignant potential of cancer is enhanced in DI-E-RAMP2-/- mice owing to compensatory activation of the AM-RAMP3 system and an increase in PDPN-positive malignant CAFs. In contrast, in RAMP3-/- mice, PDPN-negative benign CAFs predominates, resulting in a decrease in cancer malignancy (Figure 5).

The AM-RAMP2 system regulates vascular homeostasis and inhibits cancer metastasis, whereas the AM-RAMP3 system is involved in the malignant transformation of cancer-associated fibroblasts (CAFs) in the cancer microenvironment and promotes cancer metastasis.

## 3. Conclusions and Future Perspectives

Table 1 summarizes information on RAMP2 and RAMP3 from the genetically engineered mice discussed in this review.

CGRP, a family peptide of adrenomedullin and a member of the calcitonin superfamily, is known to be a causative agent of migraine. Recently, antibodies against CGRP have come into clinical use as migraine drugs [29]. Small molecular weight compounds that mimic the activity of AM are reported to reduce tumor burden in a mouse model of colitis-associated colon cancer [30]. Adrecizumab, a non-neutralizing antibody against AM, is expected to improve circulatory disturbances in sepsis [31].

Our series of studies have revealed the existence of functional differentiation among RAMP sub-isoforms, which may account for the diverse physiological functions of the calcitonin superfamily, including AM. While AM and other bioactive peptides have potential clinical applications, their short half-life in blood limits their application as therapeutic agents for chronic diseases. On the other hand, RAMPs are single transmembrane proteins of relatively small molecular weight, a structural analysis of which has been accomplished by us [32,33]. Therefore, RAMPs may be expected to serve as novel therapeutic targets instead of ligands, including AM.

Further elucidation of the pathophysiological significance and mechanism of RAMPs and artificial manipulation of their activities using specific agonists and antagonists may lead to novel therapeutic strategies, not only for cardiovascular diseases but also for the inhibition of cancer metastasis and other intractable diseases.

## Figures and Tables

**Table 1:** Phenotypes of RAMP2 knockout mice and RAMP3 knockout mice.

RAMP2 Knockout Mice	RAMP3 Knockout Mice
Conventional RAMP2-/- ref. [7]	Conventional RAMP3-/- ref. [11]
Embryonic lethal at midgestation	No abnormalities in vascular development
Edema and bleeding	Abnormal lymphatic function in adult
Abnormality of endothelial cells	Enhanced postoperative lymph edema
Endothelial cell-specific RAMP2-/- ref. [8]	
Lethal around perinatal period (95%)	
Systemic edema	
Survive until adulthood (5%)	
Vasculitis within major organs	
Organ damages	
Drug-inducible endothelial cell-specific RAMP2-/-	
Enhanced cancer cell metastasis	Reduced cancer cell metastasis ref. [26]
Cardiac myocyte cell-specific RAMP2-/- ref. [13]	Slow decline in cardiac function under cardiovascular stress ref. [13]
Rapid decline in cardiac function under cardiovascular stress	Reduced cardiac lymphatic vessels
Mitochondrial dysfunction	Reduced expression of connexin 43

## Data Availability

All data discussed in this review are included in our previously published papers listed in the references.
